# Predicting ATP-Binding Cassette Transporters Using the Random Forest Method

**DOI:** 10.3389/fgene.2020.00156

**Published:** 2020-03-25

**Authors:** Ruiyan Hou, Lida Wang, Yi-Jun Wu

**Affiliations:** ^1^Laboratory of Molecular Toxicology, State Key Laboratory of Integrated Management of Pest Insects and Rodents, Institute of Zoology, Chinese Academy of Sciences, Beijing, China; ^2^College of Life Science, University of Chinese Academy of Sciences, Beijing, China; ^3^Department of Scientific Research, General Hospital of Heilongjiang Province Land Reclamation Bureau, Harbin, China

**Keywords:** ABC transporters, random forest, classify, 188D, t-SNE

## Abstract

ATP-binding cassette (ABC) proteins play important roles in a wide variety of species. These proteins are involved in absorbing nutrients, exporting toxic substances, and regulating potassium channels, and they contribute to drug resistance in cancer cells. Therefore, the identification of ABC transporters is an urgent task. The present study used 188D as the feature extraction method, which is based on sequence information and physicochemical properties. We also visualized the feature extracted by t-Distributed Stochastic Neighbor Embedding (t-SNE). The sample based on the features extracted by 188D may be separated. Further, random forest (RF) is an efficient classifier to identify proteins. Under the 10-fold cross-validation of the model proposed here for a training set, the average accuracy rate of 10 training sets was 89.54%. We obtained values of 0.87 for specificity, 0.92 for sensitivity, and 0.79 for MCC. In the testing set, the accuracy achieved was 89%. These results suggest that the model combining 188D with RF is an optimal tool to identify ABC transporters.

## Introduction

**FIGURE 1:**
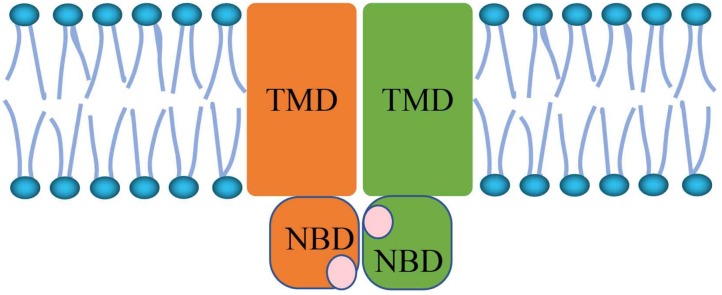
The structure of ATP-binding cassette (ABC) transporters.

The ATP-binding cassette (ABC) transporters are members of the membrane protein superfamily that translocate various molecules across extra- and intra-cellular membranes. ABC transporters are split into eight subfamilies from ABCA to ABCH. In humans, there are only seven subfamilies, designated ABCA through ABCG. Plants do not contain ABCH but instead possess ABCI (Sheps et al., 2004; Ofori et al., 2018). ABC transport proteins bind ATP and consume energy to mediate the movement of a variety of molecules across all cell membranes. As Figure 1 shows, the core architecture is a pair of conversed cytoplasmic domains: the transmembrane domain (TMD) and nucleotide binding domain (NBD) (Maqbool et al., 2015). TMDs are attached transmembrane domains that contain the ligand binding site. In most species, TMDs are composed of five to six α-helical segments (Locher, 2016). NBDs are responsible for ATP binding and hydrolysis (Locher, 2016), and conformational changes in the TMDs. The TMD amino acid sequence and topology are also different in different types of ABC transporters (Beis, 2015). The NBD domains adopt open or closed conformations by forming dimers. When NBD dimers separate, the transporter is inactive. ATP transporters possess ATPase activity when the NBD dimer conformation is closed (Gerber et al., 2008; Kadaba et al., 2008; Ward et al., 2013). During the process of conformational changes, the NBD plays the role of the transporter “engine” (Locher, 2008). Because of the important function of NBDs, the NBD domains are very conserved across different ABC transporter types.

ATP-binding cassette transporters play a very important role in many species, from simple bacteria to complex humans. In bacteria, ABC transporters include two types: ABC importers and ABC exporters. ABC importers contribute to the intake of nutrients and micronutrients by importing sugars, metal ions, and vitamins (Davidson et al., 2008; Cui and Davidson, 2011). ABC exporters are predominantly involved in exporting toxic substances and drug resistance (Seeger and van Veen, 2009; Wong et al., 2014). Bacterial ABC exporters also build lipid-linked blocks (Ruiz et al., 2008). Plant ABC transporters are involved in the exchange of secondary metabolites, coating materials, plant hormones and supportive materials. These functions are helpful to the overall development of plants (Hwang et al., 2016). In humans, most of the known functions of ABC transporters involve the transport of antigenic peptides that are relevant to biomedicine and clinical medicine (Leprohon et al., 2011). Mutations of the genes encoding ABC protein contribute to a variety of human disorders, such as cholesterol and bile transport defects, neurological disease, and cystic fibrosis. Mutations also contribute to drug resistance (Dean et al., 2001).

Many biological laboratories identified ABC transporters by artificial annotation. Pleiotropic drug resistance (PDR) transporters constitute a subfamily that belongs to the ABC superfamily. The *Nicotiana tabacum PDR* gene *NtPDR6* was identified via the Basic Local Alignment Search Tool (BLAST). Previous studies compared the *P. hybrida* genome sequence to expressed sequence tags of *N. tabacum* in the National Center for Biotechnology database using BLAST (Xie et al., 2015). They found a sequence that was similar to the *P. hybrida PDR* gene. Finally, they used the molecular biology to clone this gene and demonstrated its function. Some researchers also identified ABC transporters in monogeneans including *Gyrodactylus salaris*, *Protopolystoma xenopodis*, *Eudiplozoon nipponicum*, and *Neobenedenia melleni* and identified the transporter subfamily of each species. They identified putative ABC proteins of monogeneans by using BLASTp and screened against the putative proteins in Pfam using the HMMER web server. The server is based on protein structure and discards proteins without the conserved domains (NBD and TMD) (Caña-Bozada et al., 2019). Therefore, this method is based on homology. Putative ABC genes in the *Anopheles gambiae* genome sequence were detected using various software including GENSCAN and the HMMER package (Bateman et al., 1999) which are *ab initio* techniques. GENSCAN and HMMER are based on the Hidden Markov Model (HMM) and may be used to predict the location of genes and their exon-intron boundaries (Burge and Karlin, 1997). The accuracy of these methods needs improvement, and the experiments are time-consuming and have a tremendous cost.

With the advent of the era of big data, computational prediction based on machine learning has become a powerful approach for identifying important proteins in biology. This method does not replace biological experiments, but it improves the accuracy of prediction and provides more clues for biological experimentation. There are many examples of the application of machine learning algorithms for protein identification. A web server and software (BinMemPredict) was developed to predict membrane protein types. This approach demonstrated an accuracy of 91.2% for the identification of membrane proteins and an accuracy of 86.1% for selecting membrane protein types (Zou et al., 2013a). Pretata used a novel feature and a dimensionality reduction strategy to predict TATA binding proteins, and it achieved 92.92% prediction accuracy (Zou et al., 2016). Machine learning was also used to combine support vector machine (SVM) and PSSM distance transformation to identify DNA-binding proteins (Xu et al., 2015; Dong et al., 2019; Li Z. L. et al., 2019; Yan et al., 2019). Zou et al., proposed a model using a SVM named AOPs-SVM to identify antioxidant proteins (Jin et al., 2019).

**FIGURE 2:**
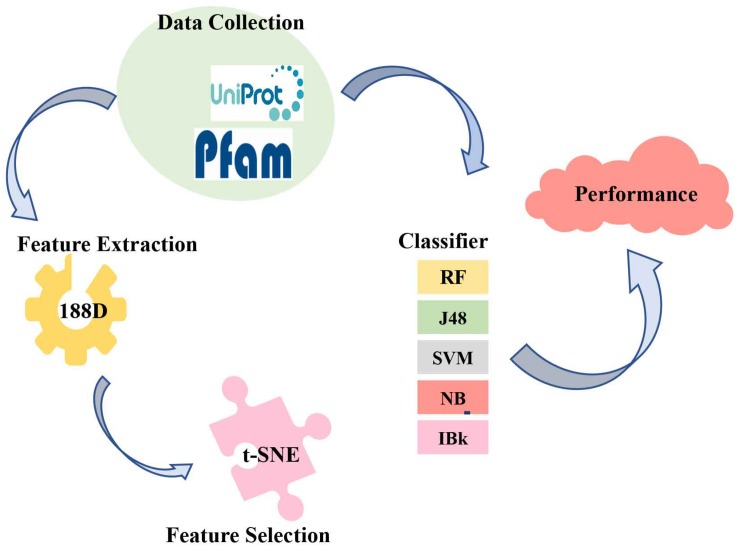
Overall Process of this study.

The present study used 188D for feature extraction and employed five classifiers to predict ABC transporters. The method of feature extraction focused on the sequence information and the physical and chemical properties. We also developed the t-SNE algorithm to visualize the features. Finally, we compared five different classifiers and revealed that random forest (RF) was the optimal model to identify ABC transporters. The overall process is shown in Figure 2.

## Materials and Methods

### Dataset

The identification of ABC transporters refers to the process of judging whether a protein is an ABC transporter. This classification problem needs two kinds of proteins. The present study used the key word “ABC transporter” to search the sequences in the Uniprot database. This search produced 1509 reviewed sequences that were used as the positive set. After obtaining the positive example set, we constructed a negative set using the following steps. We removed the families, including the above-mentioned positive sequences, from the protein family database PFAM. In these residual families, we extracted the longest protein sequence from each family as a negative sample. A total of 10661 negative example sequences was assigned to the negative data pool.

To ensure reliability of the experimental results, CD-HIT (Fu et al., 2012) was used to eliminate redundant data with a threshold of 0.6. The final dataset contained 875 positive samples and 9736 negative samples.

The positive samples and negative samples were imbalanced. To solve this problem, we randomly selected 875 negative samples from the total 9736 of negative example sequences. Therefore, the numbers of the positive samples and the negative samples were equal. This operation was repeated 10 times and we obtained 10 negative example sets. These 10 negative examples and the same positive example sets formed the 10 training sets, respectively. In every selection process, the remaining 8861 negative samples were assigned to the test set. A total of 10 test sets was obtained.

### Feature Extraction

The 188D feature extraction method was used in this study. This method extracts 188 features based on protein sequence information and physical and chemical properties. Previous researchers used the composition-position of proteins and their physical-chemical properties independently to extract protein features (Dubchak et al., 1995; Ding and Dubchak, 2001; Shen et al., 2017, 2019; Wang et al., 2017; Yu et al., 2017; Liu et al., 2018; Qiao et al., 2018; Xiong et al., 2018; Zhang et al., 2018a, b; Zou et al., 2019). In 2003, Cai et al. first combined amino acid sequences with their physicochemical properties to finish feature extraction (Cai et al., 2003). In summary, 188 features are divided into two different categories. One category consists of the amino acid composition that is expressed by 20 features. The other category consists of the physical chemical properties, including hydrophobicity, polarity, polarizability, normalized van der Waals volume, secondary structure, charge, surface tension, and solvent accessibility. The detail about the physicochemical properties is shown in Table 1.

**TABLE 1:** Three class divided according to physicochemical property.

Physicochemical property	the 1st class	the 2nd class	the 3rd class
hydrophobicity	RKEDQN	GASTPHY	CVLIMFW
Normalized van der Waals volume	GASCTPD	NVEQIL	MHKFRYW
polarity	LIFWCMVY	PATGS	HQRKNED
polarizability	GASDT	CPNVEQIL	KMHFRYW
charge	KR	ANCQGHILMFPSTWYV	DE
surface tension	GQDNAHR	KTSEC	ILMFPWYV
secondary structure	EALMQKRH	VIYCWFT	GNPSD
solvent accessibility	ALFCGIVW	RKQEND	MPSTHY

There are 20 amino acids. We calculated the respective frequency of the 20 amino acids as *n*1, *n*2, *n*3…*n*20. The feature can be expressed as:

$$\left(F\,1,F\,2,\ldots \,\ldots \,F\,20\right)=\left(\frac{n\,1}{L},\frac{n\,2}{L},\ldots \,\ldots ,\frac{n\,20}{L}\right)$$

where *F* is the feature, and *L* is the length of sequences.

Next, these 20 amino acids were divided into three types according to their physicochemical properties. The three categories included the content, distribution and the bivalent frequency, which were used to describe the physicochemical properties of proteins. We used surface tension as an example.

First, the 20 amino acids were divided into three groups (Chen et al., 2012), namely, the GQDNAHR group, KTSEC group, and ILMFPWYV group, according to their surface tensions. We calculated the content of the three groups, which are expressed as CS1, CS2, and CS3, respectively. The feature was denoted as:

$$\left(F\,21,F\,22,F\,23\right)=\left(\frac{\mathrm{CS}\,1}{L},\frac{\mathrm{CS}\,2}{L},\frac{\mathrm{CS}\,3}{L}\right)$$

For the AAs of the GQDNAHR group, the position of the first, the first and 25%, 50%, and 75% of the chain length are represented by DSij where i ranges from 1 to 3 and j ranges from 1 to 5.

$$\left(F\,24,F\,25,F\,26,F\,27,F\,28\right)=\left(\frac{\mathrm{DS}\,11}{L},\frac{\mathrm{DS}\,12}{L},\frac{\mathrm{DS}\,13}{L},\frac{\mathrm{DS}\,14}{L},\frac{\mathrm{DS}\,15}{L}\right)$$

$$\left(F\,29,F\,30,F\,31,F\,32,F\,33\right)=\left(\frac{\mathrm{DS}\,21}{L},\frac{\mathrm{DS}\,22}{L},\frac{\mathrm{DS}\,23}{L},\frac{\mathrm{DS}\,24}{L},\frac{\mathrm{DS}\,25}{L}\right)$$

$$\left(F\,34,F\,35,F\,36,F\,37,F\,38\right)=\left(\frac{\mathrm{DS}\,31}{L},\frac{\mathrm{DS}\,32}{L},\frac{\mathrm{DS}\,33}{L},\frac{\mathrm{DS}\,34}{L},\frac{\mathrm{DS}\,35}{L}\right)$$

The frequencies of occurrence of bigeminal sequences were calculated as (Zou et al., 2013b):

$$\left(F\,39,F\,40,F\,41\right)=\left(\frac{\mathrm{BS}\,1}{L-1},\frac{\mathrm{BS}\,2}{L-1},\frac{\mathrm{BS}\,3}{L-1}\right)$$

**FIGURE 3:**
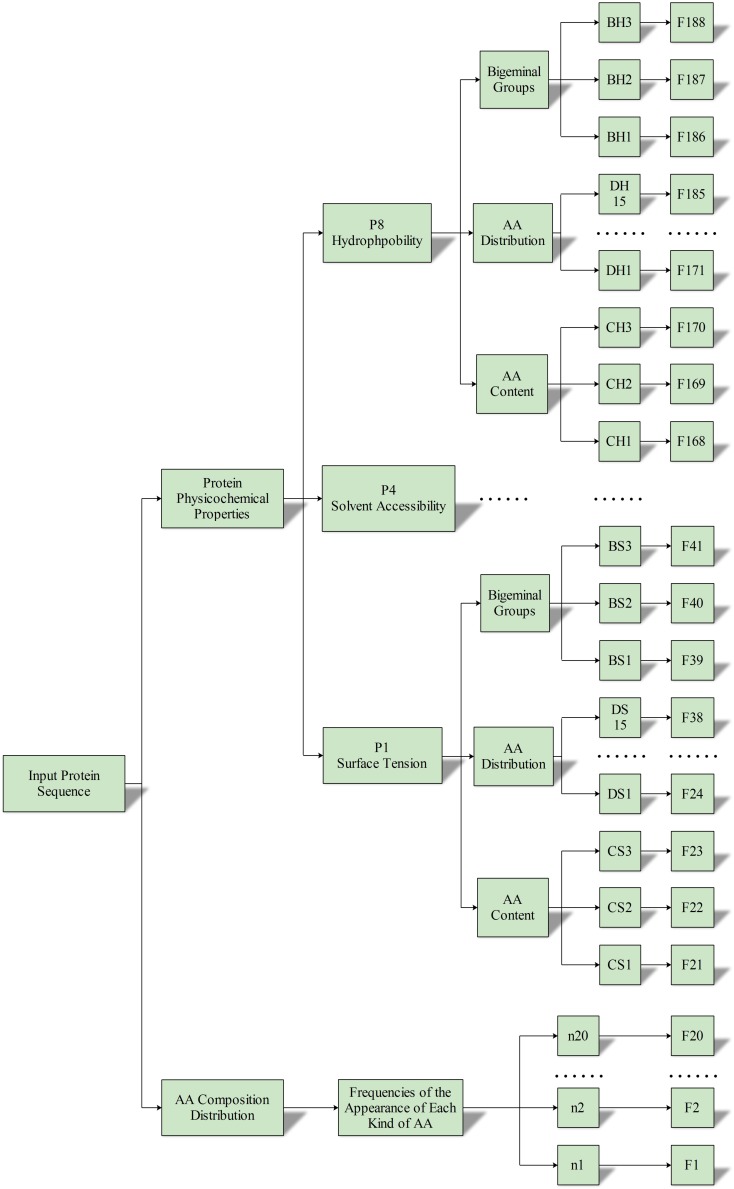
Flowchart of the 188D feature extraction method.

A total of (3 + 3 + 3× 5) = 21 feature vectors were extracted from each property, and we finally extracted all 168 (21 × 8) feature vectors from the eight physicochemical properties. In summary, the 188 (168 + 20) features were used to express the characteristics of ABC transporter protein. The process of feature extraction is illustrated in Figure 3.

### Feature Selection

t-Distributed Stochastic Neighbor Embedding (t-SNE) is a dimensionality reduction tool based on non-linear manners. t-SNE is particularly good at the visualization of high-dimensional datasets (Shao et al., 2018). The present study reduced the 188 features of protein sequences to two-dimensional features by using t-SNE. The t-SNE algorithm uses the joint probabilities to express the similarities between data points. t-SNE endeavors to minimize the Kullback–Leibler discrepancy between the joint probabilities of the low-dimensional and the high-dimensional data. t-SNE was applied to create the two-dimensional embedding in the dataset described above using the R package Rtsne. The settings of Rtsne in the present study were as follows: dims = 2, perplexity = 10, verbose = TRUE, theta = 0, max_iter = 1000, and exaggeration_factor = 8.

### Classifier

We used RF, J48, Naïve Bayes, SVM, and IBk as the classifiers. These classifiers were implemented in Weka, which contains a wide variety of machine learning algorithms based on a Java environment (Frank et al., 2004).

#### Random Forest

Random forest is an ensemble learning method that consists of many classification trees (Breiman, 2001), and it is widely used in bioinformatics research (Ding et al., 2016; Liu, 2017; Liu et al., 2017; Wei et al., 2017a, b, 2019; Zeng et al., 2017a; Yu et al., 2018; Gong et al., 2019; Lv et al., 2019; Ru et al., 2019). The “forest” is built by using bagging and random feature selection methods. The bagging method generally combines various learning models to increase the overall result. RFs are an improvement over bagged decision trees (Breiman, 1996). The details about this algorithm are described below.

First, “*M*” features were randomly selected from total “*n*” features, where *M* <<*n*. Then, we use the best split point to calculate the node “b” among the “*M*” features.

Next, by using the best split, the node was split into daughter nodes. These three steps were repeated until “l” number of nodes was reached. Finally, we constructed a “forest” by repeating the above steps for “*n*” number of times to build “*n*” number of trees. These are the pseudocode of RF creation.

After the RF model was produced, we made predictions. The test features were taken, and the codes of each randomly produced decision tree were used to predict the results. We calculated the votes for each of the predicted results. Finally, we chose the high voted prediction target as the ultimate prediction (Song et al., 2017).

#### J48

J48 implements the decision tree algorithm C4.5 (Quinlan, 1986). Ross Quinlan improved ID3 to the C4.5 algorithm in 1993. C4.5 builds decision trees from training data using information entropy. At each step, C4.5 selects an attribute of the data to effectively split into subsets. Examining the standardized information gain or the variation in entropy is the splitting criterion (Radhika and Rao, 2015; Li M. J. et al., 2019). The highest standardized information gain of an attribute is chosen to make the decision. This process recurs on each branch node. When all of the samples included in the branch nodes of the decision belong to the same class, the process is stopped (Jain et al., 2009).

#### Naïve Bayes

Naïve Bayes is an effective classifier based on the Bayesian Theorem (Cao et al., 2003). The Bayesian Theorem finds the probability of an event occurring when the probability of another event occurring is known. The Bayesian Theorem is primarily based on conditional probability, which is given in the following equation:

$$P\,\left(y|x_{1},\ldots ,x_{n}\right)=\frac{P\,\left(x_{1}|y\right)\,P\,\left(x_{2}|y\right)\,\ldots \,P\,\left(x_{n}|y\right)\,P\,\left(y\right)}{P\,\left(x_{1}\right)\,P\,\left(x_{2}\right)\,\ldots \,P\,\left(x_{n}\right)}$$

where *y* is a class variable and *x* is a dependent feature vector. *P*(*y*) is called class probability and *P*(*x*_*n*_| *y*) is called conditional probability that means the probability of *y* given *x*_1_,……,*x*_*n*_. The formula above may be expressed as:

$$P\,\left(y|x_{1},\ldots \,x_{n}\right)=\frac{P\,\left(y\right)\,\prod \begin{array}{c} n\\ i=1 \end{array}\,P\,\left(x_{i}|y\right)}{P\,\left(x_{1}\right)\,P\,\left(x_{2}\right)\,\ldots \,P\,\left(x_{n}\right)}$$

We can remove the denominator because it remains constant for a given input:

$$P\,\left(y|x_{1},\ldots ,x_{n}\right)\propto P\,\left(y\right)\,\prod \begin{array}{c} n\\ i=1 \end{array}\,P\,\left(x_{i}|y\right)$$

When this formula is applied to a Naïve Bayes, we get the probability of given features for all possible values of the class variable y and select the outcomes with maximum probability. This value may be expressed as:

$$\hat{y}={\mathrm{argman}}_{y}\,P\,\left(y\right)\,\prod \begin{array}{c} n\\ i=1 \end{array}\,P\,\left(x_{i}|y\right)$$

#### Support Vector Machine

The SVM is a supervised machine learning algorithm based on the structural risk minimization principle from statistical learning theory. Vapnik first introduced this algorithm in 1995 (Mohammad and Nagarajaram, 2011). In this algorithm, every data point was plotted as a dot in n-dimensional spaces (where n is the number of samples’ features). Then, we find an optimal hyperplane that differentiates the two classes very well. This hyperplane can maximize the margin between the two classes, and support vectors define the hyperplane. SVM has been applied to many tasks in bioinformatics (Wei et al., 2014, 2016, 2018; Ding et al., 2017; He et al., 2018; Zou et al., 2018; Fang et al., 2019; Liu and Li, 2019; Liu et al., 2019; Zeng et al., 2019b, c; Zhang M. et al., 2019; Zhang X. et al., 2019; Zhu et al., 2019).

#### IBk

The IBk is a machine learning classifier based on the k-nearest-neighbor algorithm. “Feature similarity” is used to predict the values of new data points in the K-nearest-neighbor algorithm. For implementing this algorithm, we choose training and testing data as datasets. Then, we choose an integer as “K.” We use various methods such as Euclidean, Manhattan or Hamming, to calculate the distance between the test data and each line of training data. We sorted each line of training data in increasing order based on the distance value and choose the top K lines from the sorted array. Finally, we assigned the test point to a class based on the most frequent class of these rows.

### Prediction System Assessment

The present study used some common evaluation indicators, including the total prediction accuracy (ACC), sensitivity (SN), specificity (SP), and Matthews’ correlation coefficient (MCC) (Matthews, 1975; Yu et al., 2015; Wei et al., 2017c; Zeng et al., 2017b, 2019a; Jia et al., 2018; Hong et al., 2019; Shan et al., 2019; Zhang et al., 2019a, b). These indicators are expressed as follows:

$$\mathrm{ACC}=\frac{\mathrm{TP}+\mathrm{TN}}{\mathrm{TP}+\mathrm{TN}+\mathrm{FP}+\mathrm{FN}}$$

$$\mathrm{SN}=\frac{\mathrm{TP}}{\mathrm{TP}+\mathrm{FN}}$$

$$\mathrm{SP}=\frac{\mathrm{TN}}{\mathrm{TN}+\mathrm{FP}}$$

$$\mathrm{MCC}=\frac{\mathrm{TP}\times \mathrm{TN}-\mathrm{FP}\times \mathrm{FN}}{\sqrt{\left(\mathrm{TP}+\mathrm{EN}\right)}\left(\mathrm{TP}+\mathrm{FP}\right)\,\left(\mathrm{TN}+\mathrm{FP}\right)\,\left(\mathrm{TN}+\mathrm{FN}\right)}$$

where *TP*, *TN*, *FP*, and *FN* express the rates of true positives, true negatives, false positives, and false negatives, respectively.

We also used receiver operating characteristic (ROC) curves and the area under the curve (AUC) to judge the performance of each classifier. The ROC curve is used to choose some better classifiers that maximize the true positives and minimize the false positives. Its abscissa is the false positive rate (FPR), and its ordinate is the true positive rate (TPR). We plotted the ROC curves in R. AUC is the area under the ROC curve. Generally, the higher the AUC number, the better the classifier.

## Results and Discussion

### t-SNE Visualization of the Feature Extracted by 188D

**FIGURE 4:**
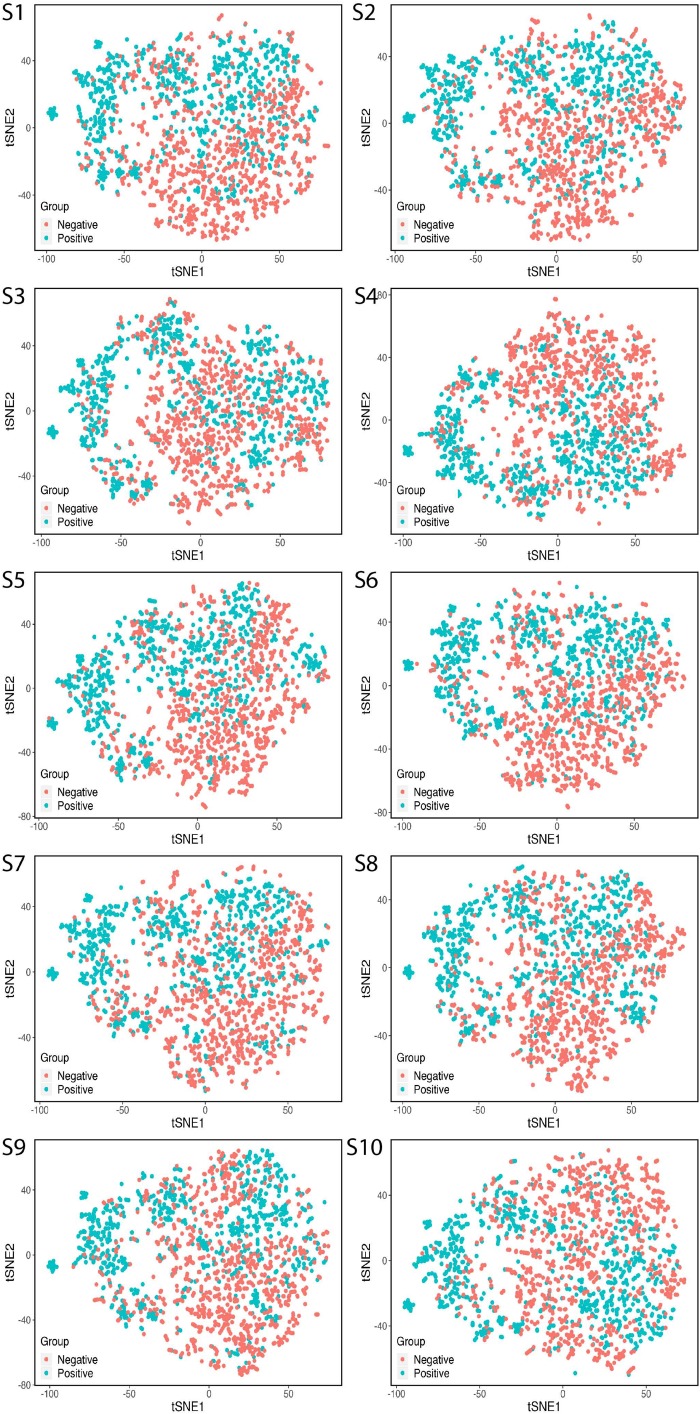
Display of the training features by t-SNE. S is the abbreviation for sample.

To examine whether the high-level features extracted by 188D had the prediction power and were generalizable, we visualized the features for the training set by applying t-SNE (Figure 4). We visualized 1750 proteins including 875 positive samples and 875 negative samples. All 10 training sets were visualized by t-SNE. Each sample had 188 features. t-SNE mapped the 188 features based on two principal features and minimized the information loss during dimension. As the figure shows, the various protein classes were almost separated clearly. The process suggested that 188D extracted representative features, and the samples were split by using t-SNE. Therefore, the classifier exhibited a sufficient performance based on these features.

### Performance of Different Classification Algorithms

To evaluate the performance of different classifiers on our data set, we used 10-fold cross-validation to select the optimal parameters in the training set by implementing WEKA. The excellent parameters in RF were obtained and evaluated on the test set. We repeated the entire process 10 times to ensure the accuracy of the experimental results.

**FIGURE 5:**
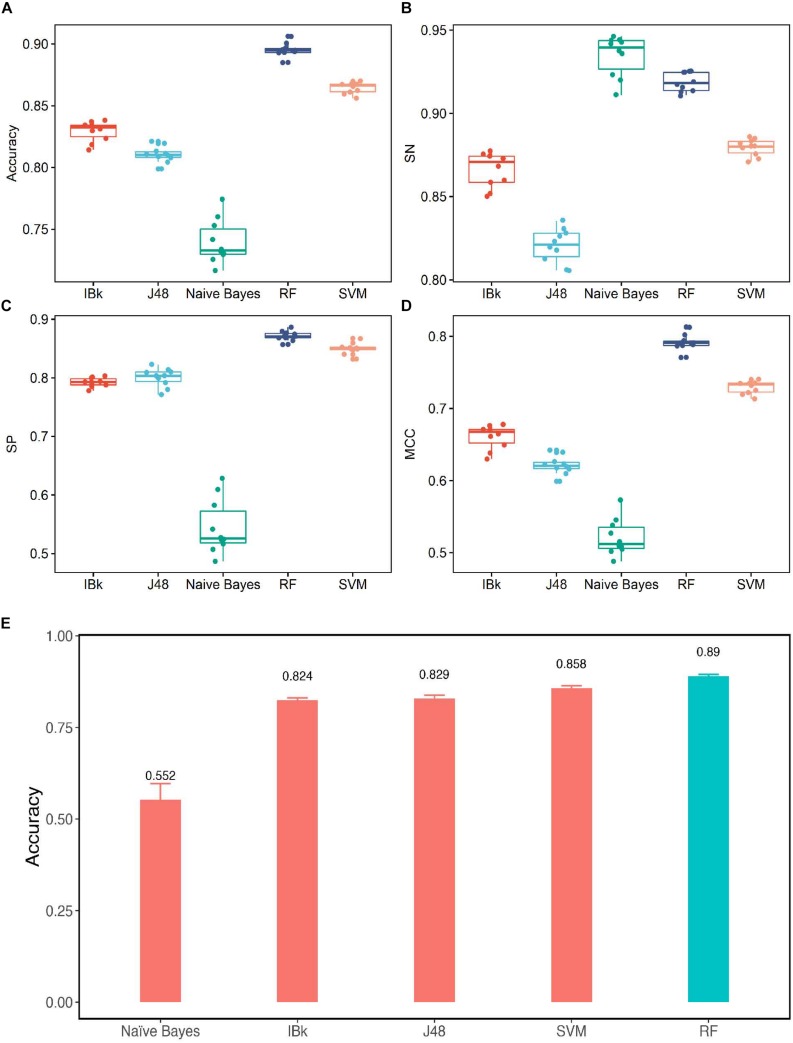
Performance of different classifier. **(A)** Accuracy comparison for the training sets by using all the classification methods. **(B)** Sensitivity comparison for the training sets using all of the classification methods. **(C)** Specificity comparison for the training sets by using all of the classification methods. **(D)** Matthew’s correlation coefficient comparison for training sets by using all the classification methods. **(E)** Accuracy comparison for the testing sets by using all of the classification methods.

The performances of different classifier models on the training set and testing sets are shown in Figure 5. For the training set, we used the total prediction accuracy, *SN*, *SP*, and *MCC* as the evaluation indicators. The weighted average of these results was used. The accuracy is shown in Figure 5A. The accuracy of RF was 0.8954, and the accuracy of Naïve Bayes was only 0.7397. Surprisingly, for the sensitivity indicator (Figure 5B), Naïve Bayes gave the best performance, and RF was close behind. The highest sensitivity score was 0.9346, and the second sensitivity score was 0.9189. Sensitivity measures the proportion of positives that were correctly identified. This result suggests that Naïve Bayes would recognize the positive samples. However, its specificity score (0.5448) was very poor. This result indicates that SVM occurred at the expense of specificity for higher sensitivity. The specificity scores of Naïve Bayes, IBk, J48, SVM, and RF were 0.5448, 0.7925, 0.8007, 0.8499, and 0.8718, respectively, as shown in Figure 5C. Obviously, the RF classifier showed the best specificity. According to the data shown in Figure 5D, the *MCC* value of RF is higher than the MCC value of the other algorithm. We achieved an MCC score of 0.7915 using the RF model. However, the lowest *MCC* value was only 0.521.

**FIGURE 6:**
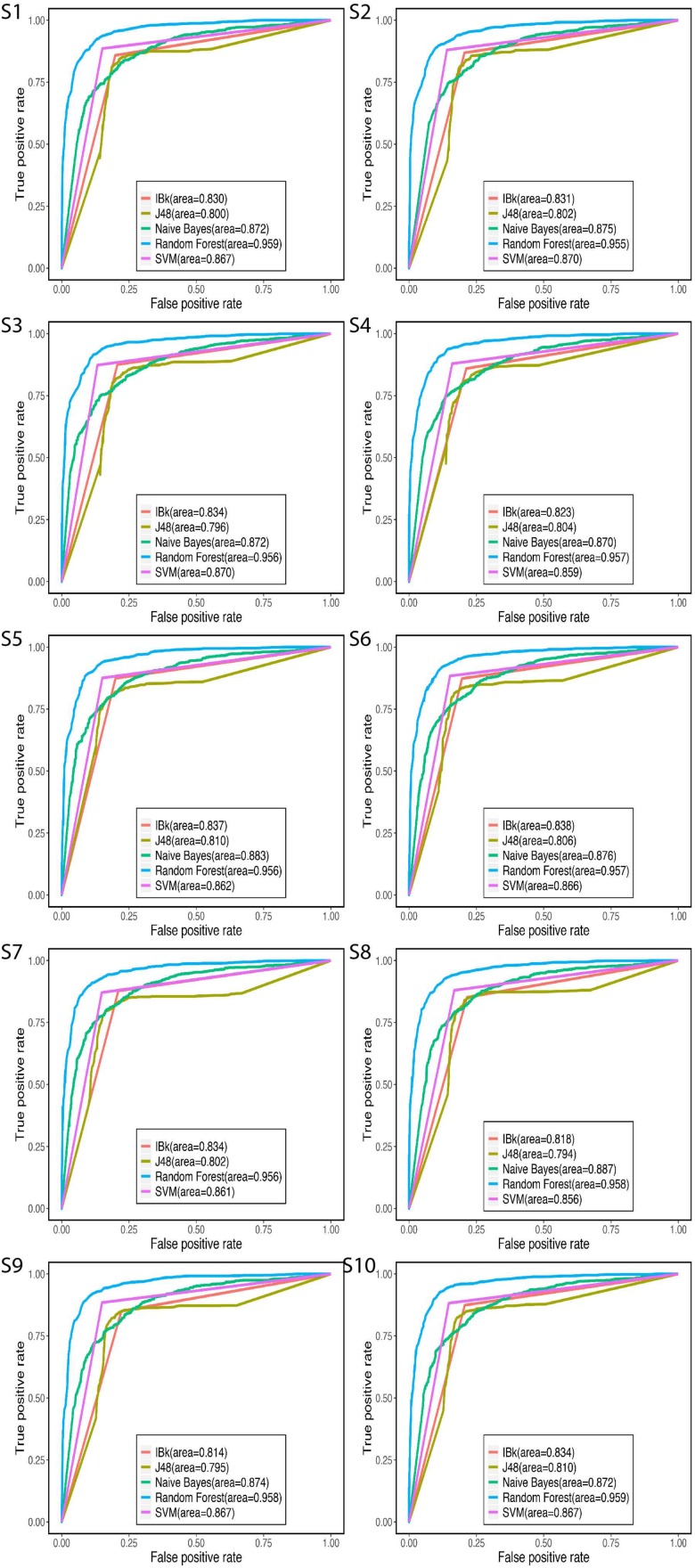
ROC curve to compare difference classifiers. S is the abbreviation for sample.

Receiver operating characteristic curves provide a useful approach to compare different classifiers. The performance of all classifiers in ROC plots is shown in Figure 6. The five classification models used in 10 randomly selected training sets performed differently. RF covered the maximum AUC in all training sets followed by Naïve Bayes, SVM, IBk, and J48. All of the AUC values of RF exceeded 95% in 10 training sets.

The testing set was used to test the models mentioned above. As the Figure 5E shows, except for Naïve Bayes, the accuracy values of the remaining classifiers exceeded 80%, and the accuracy score of RF reached 89%.

All of these indicators demonstrate that RF gives the best performance, and Naïve Bayes is the worst classifier. The RF classifier was considered the optimal classifier for prediction ABC transporter proteins in the dataset.

On the basis of the analysis above, we may draw a conclusion that the optimal strategy of identifying ABC transporter is using 188D as feature extraction method and RF as classifier.

## Data Availability Statement

Publicly available datasets were analyzed in this study. This data can be found at Uniprot: https://www.uniprot.org/uniprot/?query=ATP-binding+cassette+transporter&sort=score and https://github.com/Jenny-Jason/Predicting-ABC-transporters.

## Author Contributions

Y-JW conceived and designed the project. RH and LW performed the experiments and wrote the manuscript.

## Conflict of Interest

The authors declare that the research was conducted in the absence of any commercial or financial relationships that could be construed as a potential conflict of interest.
